# Effect of gummy candy containing ubiquinol on secretion of saliva: A randomized, double-blind, placebo-controlled parallel-group comparative study and an in vitro study

**DOI:** 10.1371/journal.pone.0214495

**Published:** 2019-04-03

**Authors:** Ryoko Ushikoshi-Nakayama, Koufuchi Ryo, Tomoe Yamazaki, Mie Kaneko, Tomoko Sugano, Yumi Ito, Naoyuki Matsumoto, Ichiro Saito

**Affiliations:** Department of Pathology, Tsurumi University School of Dental Medicine, Yokohama, Japan; Eberhard-Karls-Universitat Tubingen Medizinische Fakultat, GERMANY

## Abstract

A randomized, double-blind, placebo-controlled, parallel-group comparative clinical study was conducted to examine the effects of ubiquinol (the reduced form of Coenzyme Q10) on secretion of saliva. This interventional study enrolled 40 subjects aged 65 years or younger who were healthy, but noted slight dryness of the mouth. Subjects were randomized with stratification according to gender and age to ingestion of gummy candy containing 50 mg of ubiquinol or placebo twice daily for 8 weeks. At the end of study, along with a significant increase of the CoQ10 level in saliva (p = 0.025*, d = 0.65), there was a significant increase of the saliva flow rate (p = 0.048*, d = 0.66) in the ubiquinol candy group (n = 18; 47.4±6.2 years; 6 men and 12 women) compared to the placebo group (n = 20; 52.2±7.7 years; 4 men and 16 women). The strength of the stomatognathic muscles was not significantly enhanced by ingestion of ubiquinol candy. Compared with baseline, significant improvement of the following four questionnaire items was observed in the ubiquinol group at the end of the study: feeling tired (p = 0.00506, d = −0.726), dryness of the mouth (p = 0.04799, d = −0.648), prone to catching a cold (p = 0.00577, d = −0.963), and diarrhea (p = 0.0166, d = −0.855). There were no serious adverse events. An in vitro study revealed that ubiquinol stimulated a significant and concentration-dependent increase of ATP production by a cell line derived from human salivary gland epithelial cells (p<0.05), while 1 nM ubiquinol significantly suppressed (p = 0.028) generation of malondialdehyde by cells exposed to FeSO_4_-induced oxidative stress. These findings suggest that ubiquinol increases secretion of saliva by suppressing oxidative stress in the salivary glands and by promoting ATP production.

**Trial Registration**: UMIN-CTR UMIN000024406.

## Introduction

Xerostomia due to decreased secretion of saliva has various underlying causes, including age-related diseases and associated polypharmacy, age-related loss of perioral muscle strength, menopausal disorders, and mental stress. Not only oral pain, but also halitosis and other symptoms associated with xerostomia can have a significant impact on the quality of life [1, 2]. In recent years, the number of patients complaining of xerostomia (dry mouth) has increased, and it has been reported that impairment of the oral environment due to reduced secretion of saliva can lead to aspiration pneumonia and other diseases [3]. Thus, reduced secretion of saliva due to aging of society or stress is one of the health problems facing developed countries [4, 5].

It has been reported that 25% of the population have symptoms associated with dry mouth [6], with the international average rate of such symptoms being 28.5% among elderly individuals aged ≥ 65 years and increasing to 45% among elderly persons in aged care facilities [7]. These reports suggest that diseases related to aging may exacerbate dry mouth symptoms [8]. Current first-line treatment is drugs such as muscarinic receptor agonists that promote secretion of saliva, but adverse reactions are frequent, including sweating, polyuria, and diarrhea. Moreover, these drugs are often contraindicated in patients with cardiac or respiratory disorders [1], so use is mainly restricted to patients with dry mouth due to Sjogren’s syndrome. Some new therapies and preventive methods for dry mouth have been reported [9], including use of the antioxidant N-acetylcysteine. We have been investigating effective methods for the management of dry mouth [10–13], and we previously reported that the reduced form of ubiquinol improves dry mouth symptoms in patients with Sjogren’s syndrome [14].

It has been suggested that oxidative stress is one of the causes of age-related diseases [15, 16]. Ubiquinol has antioxidant activity and also stimulates ATP production [17, 18], suggesting that promotion of saliva secretion by ubiquinol in previous studies may have been attributable to these two effects. Ubiquinol is synthesized by humans, but its production has been shown to decrease with aging [17], and this decrease of ubiquinol is possibly associated with reduced secretion of saliva. Therefore, it is possible that maintaining a higher ubiquinol level after middle age might prevent dry mouth. Accordingly, we conducted a clinical study in healthy individuals (with a subjective feeling of slight dryness of the mouth) to evaluate the preventive effect of ubiquinol on dry mouth. We also carried out an in vitro study using a cell line derived from human salivary gland epithelium to clarify the mechanisms by which ubiquinol may influence the secretion of saliva.

## Materials and methods

### Subjects and study design

To evaluate the effect of a gummy candy containing ubiquinol on dryness of the mouth, this study enrolled healthy individuals aged < 65 years with symptoms such as a mild sensation of dryness or reduced moistness in the mouth. The subjects had symptoms such as a mild sensation of dryness or reduced moistness in the mouth, but no clear diagnosis of xerostomia even though the saliva flow rate was < 2g / 2 min in the Saxon test. Exclusion criteria were persons taking any medications and persons who may have had difficulty chewing the gummy candy because they were found to have very few teeth during the screening examination performed by the investigator or subinvestigator.

A total of 40 individuals living in Tokyo or Kanagawa Prefecture who met the above-mentioned eligibility criteria (details are given in the protocol registered with UMIN) were enrolled. This was a randomized, double-blind, placebo-controlled, parallel-group comparative study that was performed in conformity with CONSORT 2010 (S1 File). All participants provided written consent after receiving an explanation about the purpose and methods of the study. This study was approved by Tsurumi University School of Dental Medicine Ethics Review Committee (approval number: 1428) and followed the principles of the Declaration of Helsinki.The study protocol is included in the supporting information file (S2 File).

**Fig 1:**
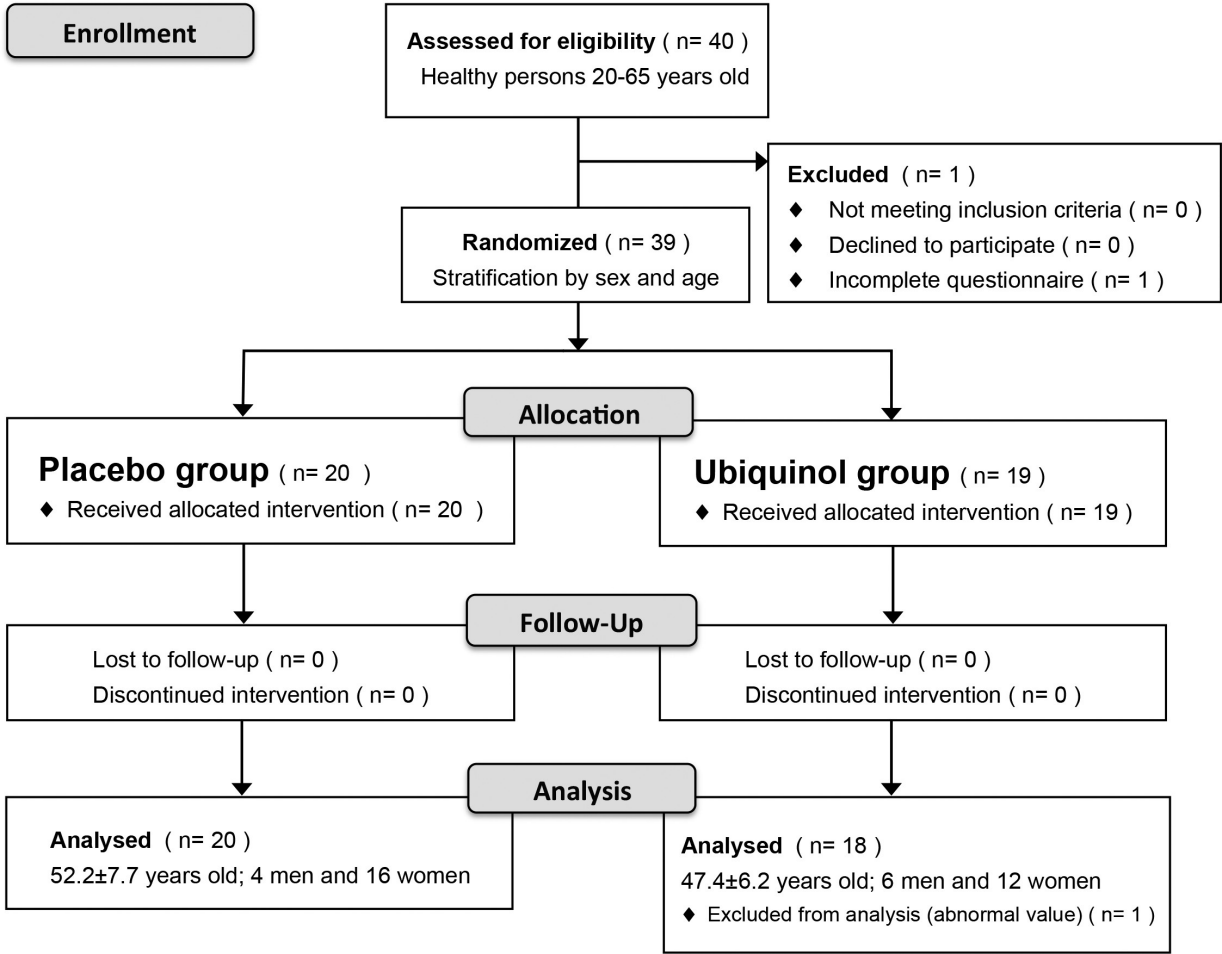
Clinical trial flow diagram.

The study was conducted between November 1, 2016 and February 25, 2017 (from enrollment to follow-up). Each phase of the study and the subject assignments are indicated in the flow diagram shown in Fig 1 There were no changes to the methods after the study started.

One of the 40 candidate subjects was excluded for not completing the questionnaire before the start of treatment. The remaining 39 subjects were randomly allocated to 2 groups with stratification according to gender and age by the study director. One group was designated as the “ubiquinol group” and the other as the “placebo group” by clerical staff independent of the study. Subjects allocated to the “ubiquinol group” ingested gummy candies containing ubiquinol, whereas subjects allocated to the “placebo group” ingested gummy candies without ubiquinol. None of the subjects dropped out after initiation of the study, but one female subject with an abnormally high salivary CoQ10 concentration (48,000 μg/ml) prior to treatment was excluded from analysis because of being a significant outlier by Grubbs’ test (P < 0.05, two-sided). Therefore, the final analysis population comprised 20 subjects in the placebo group (52.2±7.7 years old; 4 men and 16 women) and 18 subjects in the ubiquinol group (47.4±6.2 years old; 6 men and 12 women).

The test substance was a gummy candy containing 50 mg of ubiquinol, and its composition is shown in Table 1. Participants ingested 2 ubiquinol-containing candies (containing 100 mg of ubiquinol) or 2 placebo candies (identical in composition except for no ubiquinol) once a day after breakfast for 8 weeks. To prevent damage due to oxidation or photolysis, the gummy candies were stored in aluminum bags. Placebo gummy candies and gummy candies containing ubiquinol were placed into bags without any indication of the contents on the label by the person in charge of treatment assignment. Ubiquinol is tasteless and odorless, while the other components of the candies were the same and both types of candy were made to be identical in terms of color, size, taste, texture, and flavor. The assignment list was sealed and kept by the person in charge of study dug assignment, and it was not opened before evaluation of all items and statistical analyses were completed. As specified in the registered protocol, if a subject forgot to ingest the daily dose of two gummy candies, the subject could take a double doses (four candies) on the next day. This design was intended to facilitate compliance by the subjects during long-term intervention, and none of the subjects had a large amount of unused gummy candies at the end of the study.

**Table 1:** Composition of the candies.

Ubiquinol-containing candy	Placebo candy
Multirole syrup	Multirole syrup
Multirole	Multirole
Polydextrose	Polydextrose
Gelatin	Gelatin
**Ubiquinol**	**Vegetable oil**
Glycerin	Glycerin
Ascorbic acid	Ascorbic acid
Acidulant	Acidulant
Pectin	Pectin
Flavor	Flavor
Coloring (Carotene)	Coloring (Carotene)
Glazing agent	Glazing agent
Steviol glycosides	Steviol glycosides
Green tea extract	Green tea extract

The primary evaluation items were the saliva flow rate and salivary CoQ10 level. In addition, the repeated saliva swallowing test, bite force test, and a questionnaire survey of symptoms were conducted before and after study treatment. No changes were made to these items for evaluation after completion of the study.

### Saliva flow rate measurement

The Saxon test was performed to measure saliva flow stimulated by chewing [19]. Secretion of saliva was measured when the subject chewed a 7.5 × 7.5 cm square of sterile gauze with a mean dry weight of 1.7 g (pure sterilized gauze, size M, Tamagawa Eizai Co., Ltd., Tokyo, Japan) for 2 minutes at a rate of once per second. The gauze was weighed before the test, and the saliva flow rate was determined by subtracting the dry weight from the wet weight measured after chewing.

### Determination of the salivary Coenzyme Q10 (CoQ10) concentration

Resting saliva (1–1.5 ml) was collected from each subject into a 2-ml sample tube before and after the study intervention, and the samples were stored at −80°C until analysis. The total salivary CoQ10 level was measured by Kaneka Techno Research Co., Ltd. using liquid chromatography with tandem mass spectrometry (LC/MS/MS) [20]. In brief, 0.7 mL of the extraction solvent (isopropanol containing 2 mg/mL of 1, 4-benzoquinone and 20 ng/mL of CoQ9) was added to 0.1 mL of saliva and mixed. After centrifugation, the supernatant was filtered through a membrane filter and used as the sample for LC/MS/MS, which was performed using an AB Sciex Triple Quad 5500 system (AB Sciex, Framingham, MA, USA).

### Repeated saliva swallowing test

The repeated saliva swallowing test (RSST) was performed to evaluate swallowing [21, 22]. With the subject in the sitting position, palpation of the laryngeal prominence and hyoid bone was performed to detect the shift of these structures during deglutition, and the number of times saliva was swallowed during a 30-second period was measured.

### Bite force measurement

Bite force was measured with an occlusal force meter (Nagano-keiki, Co., Ltd., Tokyo, Japan) using a method described previously [23], which was placed between the teeth. The maximum value of three measurements was recorded.

### Assessment of symptoms

Before and after the study treatment, subjects were asked a total of 78 questions (with responses on a 5-point scale), including 26 questions about oral symptoms and 53 questions regarding physical and mental symptoms [24] (S3 File). The responses were analyzed.

### Statistical analysis

Results are presented as the mean ± standard deviation. A sample size test based on a noncentric t-distribution was performed using data on saliva secretion after the intervention in the ubiquinol group and placebo group. The correlation between age and the amount of saliva secretion before initiation of the study was calculated. To perform analysis of measured parameters, Student's t-test or the paired t-test was used for parametric data. When data were nonparametric, the Mann-Whitney U test was used for paired groups and the Wald-Wolfowitz runs test [25] was employed for independent groups. The effect size (mean difference between the 2 groups) was assessed by calculating Cohen's d value [26]. Analyses were performed with StatPlus:mac (AnalystSoft Inc., CA., USA) and Psychometrica statistical software [27]. All numerical data used for analysis are included in the supporting information (S4 File).

### Effect of ubiquinol on a human salivary gland cell line

#### ATP assay

The in vitro study was performed using human salivary gland (HSG) cells, an epithelial cell line derived from human submandibular intercalated duct cells [28]. Cells were seeded at 2×10^3^ /well into a 96-well clear bottomed white plate and cultured overnight, after which the medium was replaced with fresh medium containing ubiquinol (1, 10, or 100 nM) or without ubiquinol (0 nM). Then incubation was continued for another 48 hours. Next, “Cellno” ATP assay reagent (TOYO B-Net Co., Ltd., Japan) was added to the cells, and the intensity of fluorescence associated with ATP was measured with a plate reader (Wallac 1420 ARVOsx, PerkinElmer Japan Co., Ltd., Japan). Results were analyzed by one way ANOVA and a post-hoc Tukey-Kramer test.

#### Lipid peroxidation assay

Confluent HSG cells in 10-cm dishes were cultured for 1 hour after exchanging the medium for medium containing ubiquinol (1, 10, or 100 nM) or 100 μM sodium ascorbate as a control. Then FeSO_4_ was added at a final concentration of 100 μM, and incubation was continued for 30 minutes [29]. Malondialdehyde (MDA) was measured by the TBARS method using a Malondialdehyde Assay Kit (Northwest Life Science Specialties, USA) [30, 31]. In brief, treated cells were harvested in 300 μl of MDA assay buffer and were sonicated using an ultrasonic crusher (Biorupter UCD-250, BM Equipment, Tokyo, Japan) (10 sec × 25 times). Then the sonicated cells were centrifuged at 15,000×g for 5 minutes, and the supernatant was used for measurement. Data were analyzed by the 3rd derivative method to obtain MDA concentrations [31], and statistical analysis was performed by Welch’s t-test (one-sided).

#### Carbonylated protein assay

HSG cells cultured for 1 hour in the presence or absence of ubiquinol were subjected to oxidative stress by incubation with 20 mM H_2_O_2_ for 30 minutes [29]. Then oxidation of proteins was analyzed by using an OxyBlotTM Protein Oxidation Detection Kit (Chemicon, USA). Treated cells were sonicated in radioimmunoprecipitation buffer and centrifuged to obtain the supernatant, which was used to assess carbonyl group derivatization by adding 2,4-dinitrophenylhydrazine (DNPH) at a final concentration of 10 mM. Samples were resolved by SDS-PAGE with 12% polyacrylamide gel and transferred to PVDF membranes using a semi-dry blotting system (Bio-Rad Laboratories, Inc., USA). Carbonylated proteins were detected with an anti-DNP antibody (supplied with the kit), and the intensity of luminescence associated with DNP was quantified by using Image J software [32].

## Results

### Clinical study

**Fig 2:**
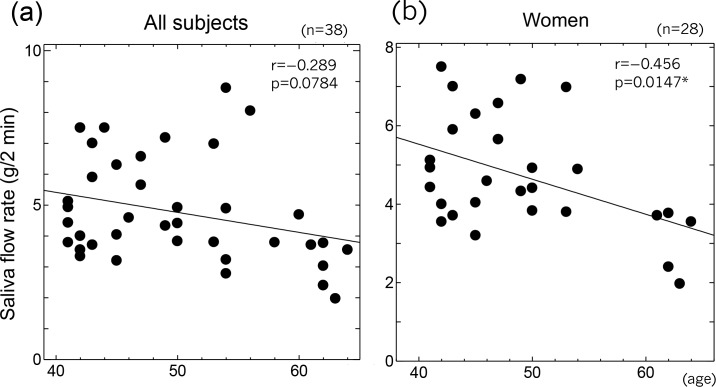
Correlation between age and the saliva flow rate. (a) Overall analysis. Age and the saliva flow rate showed a weak negative correlation that was not significant. (b) Analysis of female subjects. Age and the saliva flow rate showed a significant negative correlation in women.

There were no significant differences of the saliva flow rate, salivary CoQ10 level, or RSST results between the ubiquinol group and the placebo group before the start of study treatment, but there was a significant difference of baseline bite force on the left side (Table 2). The saliva flow rate decreased with age, although the change was not statistically significant (r = -0.289, p = 0.0784). However, analysis of the female subjects (n = 28) revealed a significant negative correlation between the saliva flow rate and age (r = -0.456, p = 0.0147) (Fig 2).

**Table 2:** Baseline characteristics of the subjects.

Saliva flow and oral function	Placebo group (n = 20)	Ubiquinol group (n = 18)	P value
**Saliva flow rate (g/2min)**	4.40±1.46	5.18±1.76	0.155
**Salivary CoQ10 level (μg/ml)**	84.5±92.3	105.7±243.7	0.081
**RSST**	5.35±1.30	5.89±2.38	0.084
**Bite force (right)**	0.33±0.20	0.40±0.18	0.309
**Bite force (left)**	0.33±0.21	0.43±0.20	0.006**

Values are the mean ± SD.

**: p>0.01 by the Wald-Wolfowitz Runs Test).

Except for bite force on the left side, there were no significant differences of baseline parameters between the ubiquinol group and the placebo group.

**Fig 3:**
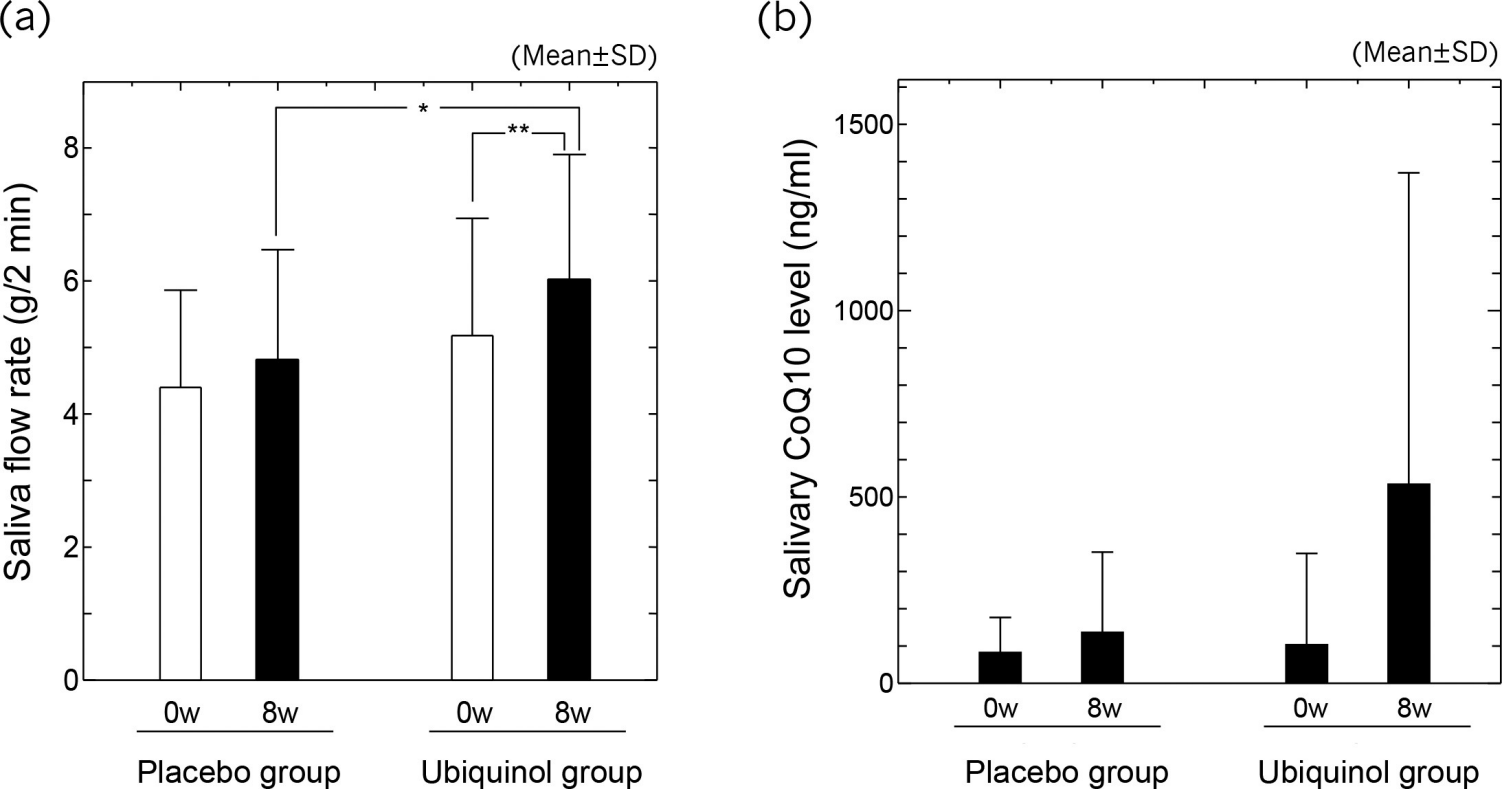
Saliva flow rate and salivary CoQ10 level before and after the study. The saliva flow rate (a) and salivary CoQ10 level (b) in the placebo group (n = 20) and the ubiquinol group (n = 18) before (0w) and after (8w) the treatment period. Values are presented as the mean ± standard deviation. Significant differences:* p <0.05, ** p <0.01.

After the treatment period, the saliva flow rate was 4.81±1.66 in the placebo group versus 6.02±1.88 in the ubiquinol group, and the saliva flow rate was significantly higher in the ubiquinol group than in the placebo group, with a moderate effect size being recognized (p = 0.048, d = 0.66) (Table 3, Fig 3(A)). Within-group comparison revealed an increase of the saliva flow rate by 16% in the ubiquinol group after treatment (p = 0.00963**, d = 0.45). No significant change of the saliva flow rate occurred in the placebo group (p = 0.130, d = 0.26) (Table 3, Fig 3(A)). Calculation based on a noncentric t-distribution showed that the required sample size was a total of 64 subjects when α = 0.05 and 1−β = 0.8, but the actual study sample size was smaller (n = 38). However, the difference between the two groups (μ1 − μ2) was 1.208 and the power of test was 81.1%, indicating that the sample size was large enough for statistical validity.

**Table 3:** Comparison of parameters between before and after the treatment period.

Saliva and oral function	Placebo		Ubiquinol		Comparison between the groups at 8 w
	0 w	8 w	0 w	8 w	
**Saliva flow rate (g/2 min)**	4.4	4.81	5.18	6.02	
	±1.46	±1.66	±1.76	±1.88	p = 0.048*
	p = 0.130		p = 0.00963**		d = 0.66
	d = 0.26		d = 0.45		
**Salivary CoQ10 level (μg/ml)**	84.5	139	105.7	536.2	
	±92.3	±213.	±243.7	±834.4	p = 0.025*
	p = 0.212		p = 0.00956**		d = 0.65
	d = 0.33		d = 0.7		
**RSST**	5.35	7	5.89	6.72	
	±1.30	±2.43	±2.38	±3.30	p = 0.163
	p = 0.00078***		p = 0.263		d = -0.097
	d = 0.77		d = 0.29		
**Bite force (right)**	0.33	0.36	0.4	0.47	
	±0.20	±0.20	±0.18	±0.18	p = 0.093
	p = 0.563		p = 0.062		d = 0.54
	d = 0.15		d = 0.36		
**Bite force (left)**	0.33	0.37	0.43	0.47	
	±0.21	±0.21	±0.20	±0.20	p = 0.0038**
	p = 0.108		p = 0.381		d = 0.49
	d = 0.19		d = 0.2		

Values are the mean ± SD. Significant differences

* p<0.05

** p<0.01.

A Cohen’s d value ≥ 0.2 is defined as a small effect size, while d ≥ 0.5 is a moderate effect size and d ≥ 0.8 is a large effect size [26].

After ingestion of gummy candies, the mean salivary level of CoQ10 showed a significant increase by approximately 5-fold in the ubiquinol group and the effect size was moderate (p = 0.00956**, d = 0.7) (Table 3, Fig 3(B)).

There was a significant difference of the RSST (moderate effect size) between before and after intervention in the placebo group (p = 0.00076***, d = 0.77). However, no significant difference of the RSST (small effect size) was seen in the ubiquinol group (p = 0.263, d = 0.29), and comparison between the two groups after intervention also revealed no significant difference (p = 0.163, d = -0.097). At the end of the study, there was still a significant between-group difference of bite force on the left side, as was noted at baseline, but there was no difference of bite force on the right side (right: p = 0.093, d = 0.54, left: p = 0.0038**, d = 0.49) (Table 3).

For the items in the symptom questionnaire, within-group comparison between before and after intervention was performed using the Wilcoxon matched pairs test and items showing p < 0.05 were investigated further to identify those with a moderate effect size (Cohen' s d < −0.5) and a 95% CI < 0. Each item in the questionnaire was scored on a 5-point scale and a higher score corresponded to more severe symptoms, so improvement was indicated when the effect size and the 95% CI were both negative values. In the ubiquinol group, comparison between before and after intervention revealed significant improvement of the following four items: “feeling tired” (p = 0.00506, d = −0.726, 95%CI = −1.366 –−0.086), “dryness of the mouth” (p = 0.04799, d = −0.648, 95%CI = −1.284 –−0.012), “prone to catching a cold” (p = 0.00577, d = −0.963, 95%CI = −1.618 –−0.309), and “diarrhea” (p = 0.0166, d = −0.855, 95%CI = −1.503 –−0.208). In the placebo group, significant improvement of the following three items was observed: “worried about halitosis” (p = 0.01128, d = −0.672, 95%CI = −1.309 –−0.035), “stomachache” (p = 0.00963, d = −0.675, 95%CI = −1.312 –−0.038), and “cannot feel happy” (p = 0.019, d = −0.768, 95%CI = −1.411 –−0.126). In both groups, none of the symptoms (questionnaire items) became significantly worse, and there were no adverse drug reactions or serious adverse events.

### In vitro study

Based on the results of the above clinical study, investigations using cultured cells was carried out to investigate the mechanism by which ubiquinol altered the secretion of saliva.

**Fig 4:**
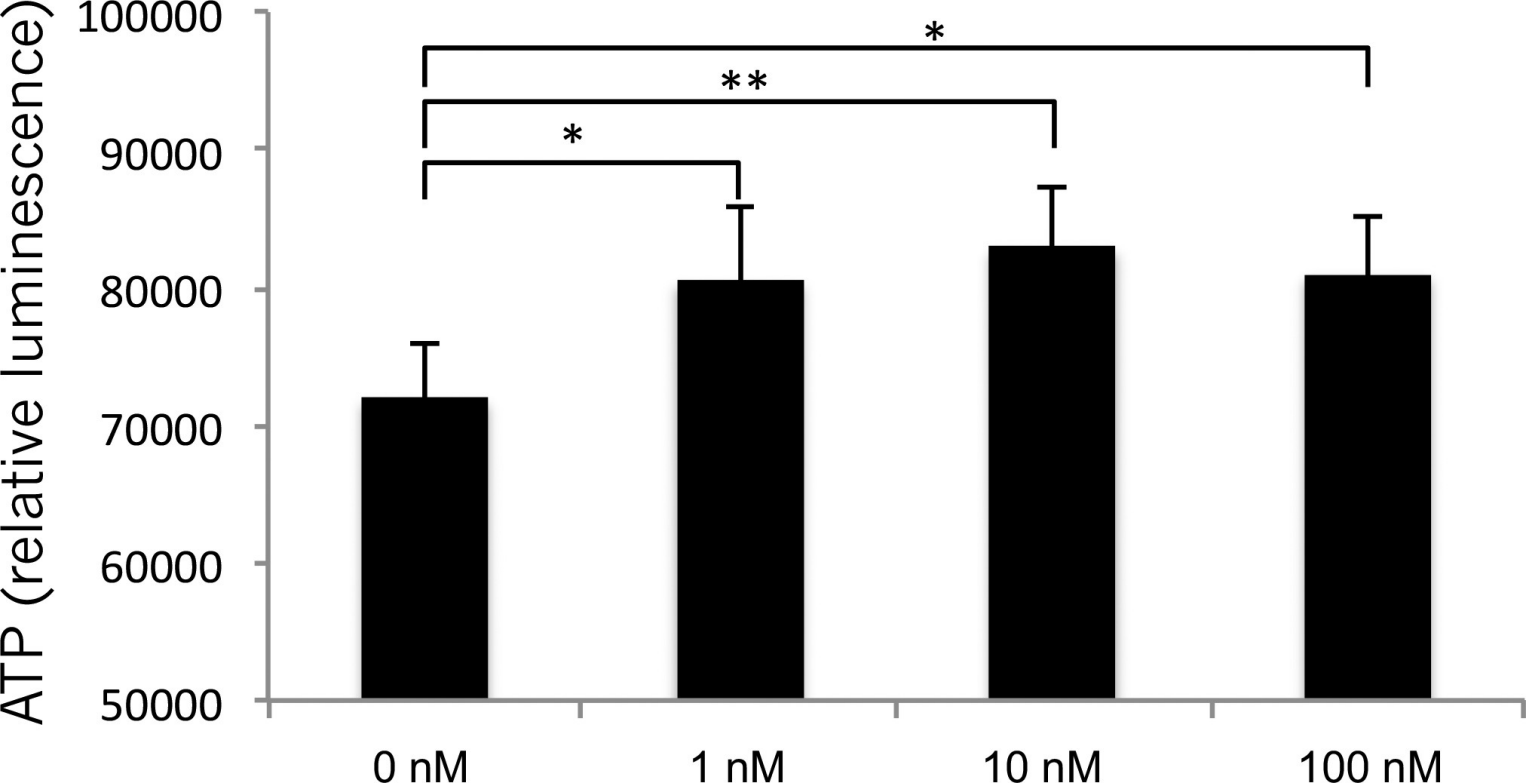
ATP production by HSG cells. ATP production by human salivary gland (HSG) cells was increased by incubation with ubiquinol in a concentration-dependent manner. Values are presented as the mean ± standard deviation (n = 4). Significant differences: * p <0.05.

Fig 4 shows the effects of ubiquinol on ATP production by HSG cells derived from salivary gland cells. The fluorescence intensity (indicating intracellular ATP) showed a significant increase in a concentration-dependent manner when HSG cells were stimulated by ubiquinol (1 nM; p = 0.058, 10 nM; p = 0.014*, 100 nM; p = 0.021*).

**Fig 5:**
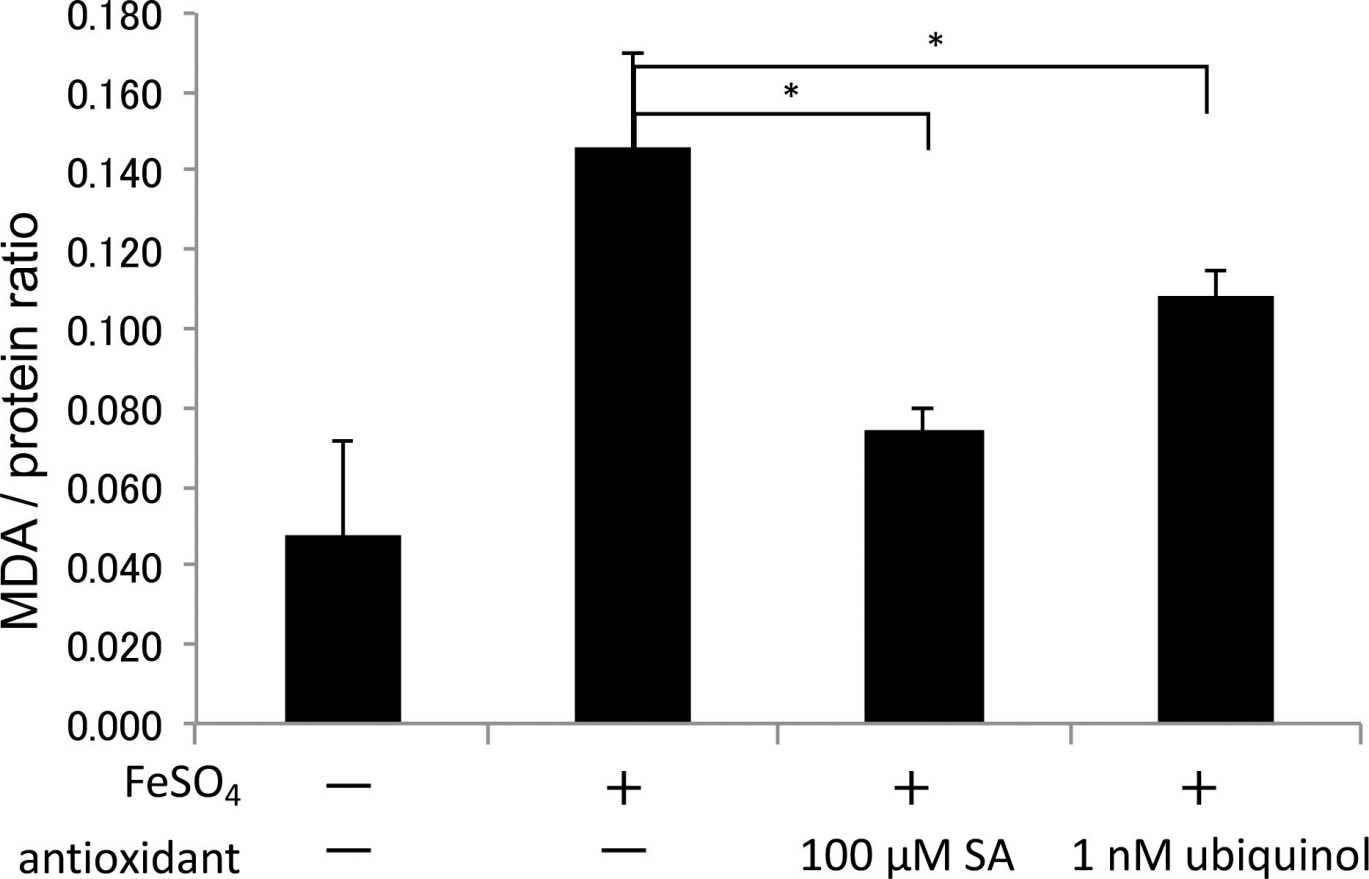
Detection of MDA (lipid peroxidation) in HSG cells. In human salivary gland (HSG) cells, lipid peroxidation induced by 100 μM FeSO_4_ was inhibited by 100 μM sodium ascorbate (SA) and 1 nM ubiquinol. Values are presented as the mean ± standard deviation (n = 3). Significant differences: * p <0.05, ** p <0.01.

When oxidative stress was induced by exposure of HSG cells to FeSO_4_, MDA increased due to lipid peroxidation, while generation of MDA was significantly suppressed in the presence of 1 nM ubiquinol (p = 0.026*) (Fig 5).

**Fig 6:**
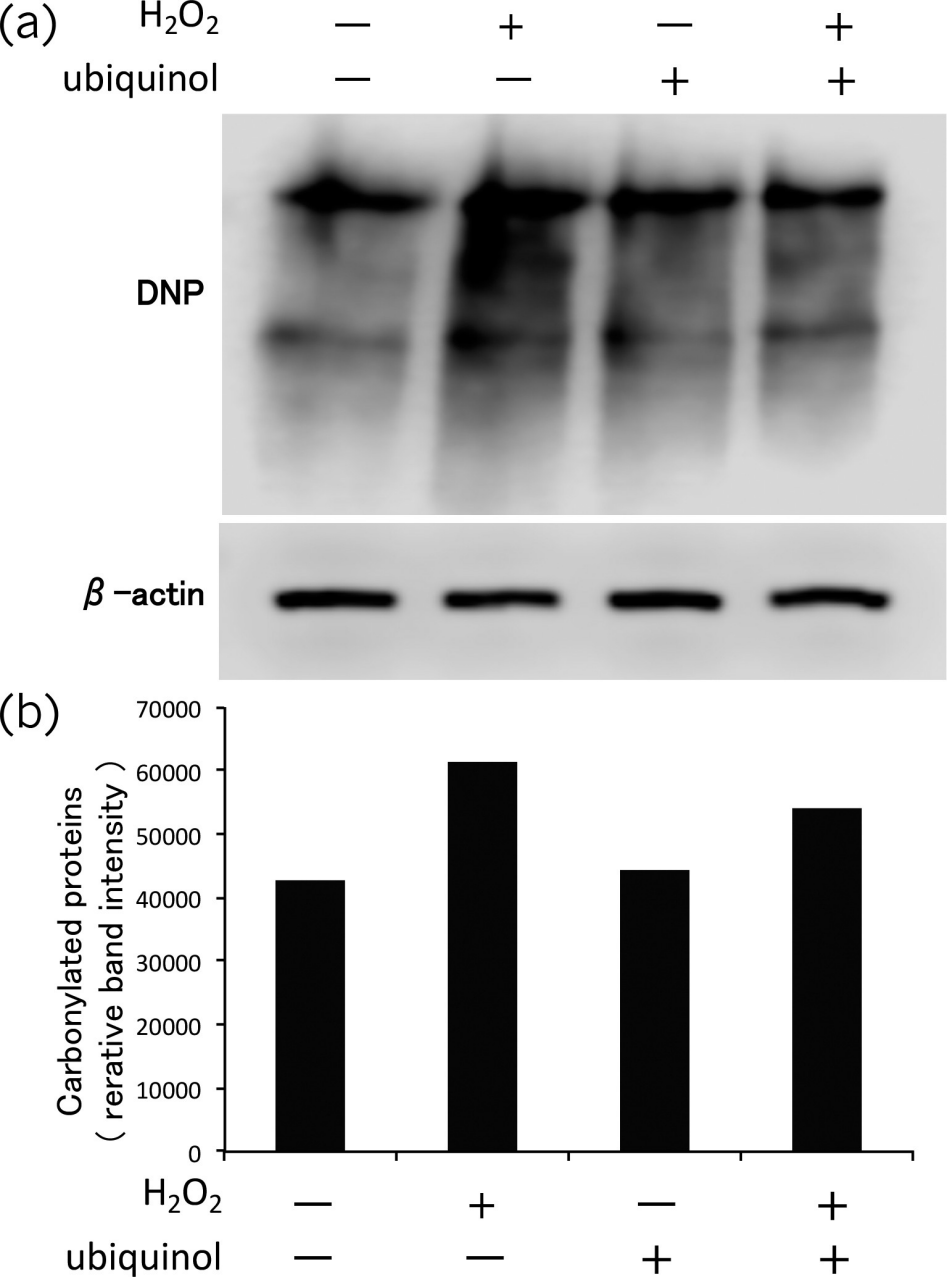
Protein carbonylation assay. Oxidation of proteins in human salivary gland (HSG) cells induced by 20 mM H_2_O_2_ was inhibited by 100 nM ubiquinol. (a) Western blots of carbonylated proteins stained with anti-DNP antibody and β-actin in the same samples. (b) The staining intensity of carbonylated protein bands was quantified by using Image J software.

Treatment of HSG cells with H_2_O_2_ induced protein oxidation (carbonylation), while this effect was suppressed by ubiquinol (Fig 6).

## Discussion

In recent years, oral disorders such as impaired secretion of saliva have been found to have a negative influence on other diseases, and oral care measures to extend healthy life expectancy are growing in importance [4, 33]. While maintaining the secretion of saliva is considered important to promote a healthy oral environment, various factors are known to reduce saliva production, including age-related diseases [6]. In particular, the decline of saliva production with aging is influenced by reduced oral and maxillofacial muscle strength, while intake of soft foods that reduces the time spent masticating or the decrease of conversation due to the widespread adoption of information technology systems are considered to exacerbate the situation [1].

The present study targeted healthy individuals who had mild symptoms such as dryness of the mouth and insufficient moistness, but did not have a diagnosis of xerostomia. Their baseline saliva flow rate was 4.77±1.66 g/2 min, which is about 6-fold higher than that previously reported in patients with xerostomia (0.75 g/2 min) [14]. Nonetheless, we observed a significant increase of the saliva flow rate after ingestion of ubiquinol-containing candy. The absolute effect size revealed by comparison between before and after the intervention was not so large in the ubiquinol group, presumably because this study was conducted in healthy individuals. However, although the number of subjects enrolled and their residential area were limited, saliva secretion was significantly increased in the ubiquinol group compared with the placebo group after the intervention, suggesting that intake of ubiquinol promoted the secretion of saliva. Accordingly, the results of this study could probably be widely generalized. We previously reported that the salivary CoQ10 level and saliva flow rate both increased when patients with xerostomia, including those with Sjogren’s syndrome, ingested ubiquinol capsules (100 mg of ubiquinol daily) [14]. In the present study, the mean salivary CoQ10 level showed a significant increase by about 5-fold after the intervention in the ubiquinol group compared with baseline (p = 0.00956, d = 0.7). This suggests that orally ingested ubiquinol is transported to the salivary glands and excreted in saliva. Sugano et al. reported that intake of ubiquinol capsules alleviated gingival inflammation in healthy individuals with mild to moderate chronic periodontitis not requiring specific treatment, with this improvement being due to increased saliva flow and higher salivary antioxidant activity [34]. In addition, application of ubiquinol to the gingivae in rats increased antioxidant activity and had a local anti-inflammatory effect [35]. These reports suggest that ubiquinol can act directly on the gingivae or via oral intake.

It is known that secretion of saliva is increased by stimulation of the stomatognathic muscles due to chewing. It was reported that ubiquinol can protect skeletal muscle against oxidative damage through its antioxidant activity and can augment skeletal muscle through promotion of muscle cell growth [36, 37]. In the present study, we expected that intake of ubiquinol would enhance stomatognathic muscle function and have a positive influence on saliva secretion, so we investigated the RSST and bite force. However, bite force showed no change from baseline in both groups. While the RSST parameters increased significantly from baseline in the placebo group, no significant changes were observed after intervention in the ubiquinol group and there were no significant differences between the two groups at the end of the study. We selected the RSST for this study because it has been recommend as a simple tool for assessment of swallowing function by the Japan Long-term Care Insurance Act and because it is a and low-risk test. Accuracy of the RSST is influenced by the participant’s cognitive ability and verbal communication ability, but there would be little effect of such factors in this study because the subjects were healthy persons. If a more accurate test than the RSST, such as the 100-ml water drinking test, had been used for evaluation, our finding that intake of ubiquinol did not influence stomatognathic muscle function may have been different. We also predicted that regular chewing of gummy candies over the long study period might strengthen the jaw muscles and promote saliva secretion. However, comparison between before and after intervention showed that RSST values only increased significantly in the placebo group, so whether chewing gummy candies can increase muscle strength remains unclear. Because gummy candies melt relatively quickly while being chewed, the candies may have had little effect on jaw muscle strength. If the participants had chewed gum or some other substance for around 10 minutes each time, the results of this study might have been different. Accordingly, we concluded that the increase of saliva secretion in the ubiquinol group was related to the effect of ubiquinol on the salivary gland rather than to stimulation of the stomatognathic muscles.

The symptom questionnaire showed significant improvement of four symptoms in the ubiquinol group: “feeling tired”, “dryness of the mouth”, “prone to catching a cold”, and “diarrhea”. The improvement of malaise / fatigue (feeling tired) in this study was consistent with past reports on improvement of chronic fatigue syndrome by CoQ10 [38], and improvement of dry mouth was probably due to increased secretion of saliva. Regarding the changes of “prone to catching a cold” and “diarrhea”, compromised immune function due to oxidative stress may have been improved by the antioxidant effect of ubiquinol and then had a preventive effect on infectious diseases. In the placebo group, there was significant improvement of three items: “worried about halitosis”, “stomachache”, and “cannot feel happy.” These changes were considered to be due to the psychological effect of participation in the study.

It is important to prevent a decline of salivary gland function in order to maintain adequate flow of saliva. Tissue damage due to oxidative stress caused by oxygen radicals, etc. has been suggested to directly impair salivary gland function, so protection against oxidative damage may be important for maintaining adequate production of saliva. Disturbance of saliva secretion is frequent in women, especially after menopause [1, 39].

In this study, the female participants were within the age range from about 5 years younger to about 5 years older than the average age of menopause, and we found that secretion of saliva decreased with age and showed a significant inverse correlation with age among females. Because female hormones have antioxidant activity [40], reduction of female hormones due to menopause may increase oxidative stress and lead to impairment of salivary gland function. We have previously evaluated the effects of various antioxidants on secretion of saliva [10–13]. Ubiquinol is a lipid-soluble antioxidant that scavenges lipid radicals and reduces oxidized vitamin E [41–44]. It was reported that ubiquinol and vitamin E act cooperatively to prevent lipid peroxidation, but ubiquinol efficiently inhibits lipid peroxidation even in the absence of vitamin E [45]. These reports suggest that ubiquinol could play an important role in preventing oxidative damage to lipids and cell membranes. In the present study, we found that addition of ubiquinol to cultures of HSG cells, a human submandibular gland epithelial cell line, suppressed lipid peroxidation initiated by FeSO_4_ and protein oxidation due to H_2_O_2_ These results suggest that ubiquinol alone (without vitamin E) is sufficient to suppress oxidative damage to salivary gland cells.

Ubiquinol has been reported to activate ATP production as a component of the mitochondrial electron transport system [46–48]. ATP is an energy source for acinar myoepithelial contraction in the salivary gland, and the ATP-dependent Na+-K+ pump (Na+-K+-ATPase) is expressed in the muscarinic receptor signaling pathway that facilitates saliva secretion, while an increase of ATP in salivary gland tissues promotes the production of saliva [49]. Accordingly, we investigated whether ubiquinol had any influence on ATP production in HSG cells. We found that production of ATP was increased by ubiquinol in a concentration-dependent manner, suggesting that ubiquinol can promote secretion of saliva by augmenting ATP production.

Taken together, these results suggest that ubiquinol suppresses oxidative damage to salivary gland cells and enhances energy production, thus increasing secretion of saliva and the saliva flow rate.

## Conclusion

Ingestion of ubiquinol-containing candy alleviated symptoms related to dryness of the mouth and increase the saliva flow rate in healthy individuals. Our in vitro experiments suggested that improvement of salivary gland secretion by ubiquinol might be related to its antioxidant activity and to promotion of mitochondrial ATP production.

## Supporting information

S1 File CONSORT checklist. (PDF)

S2 File Protocol in Japanese with English translation. (PDF)

S3 File Symptom questionnaire. (XLSX)

S4 File Data used in all analyses. (XLSX)
