# Supplementary material for: Effect of gummy candy containing ubiquinol on secretion of saliva: A randomized, double-blind, placebo-controlled parallel-group comparative study and an in vitro study
Source: PLoS One. 2019 Apr 3;14(4):e0214495. doi: 10.1371/journal.pone.0214495 (PMC6447281; doi:10.1371/journal.pone.0214495)
Supplement: S2 File — (PDF) [file pone.0214495.s002.pdf]

別紙様式１（第１０条関係）

倫 理 審 査 申 請 書

平成 28 年 10 月 5 日

鶴見大学学長　　殿

実施責任者
所 属　歯学部病理学講座
職 名　講 師
氏 名　梁 洪淵　㊞

*受付番号

１．審査対象
研究デザイン（該当を○で囲む）
①介入研究／観察研究
②侵襲を伴う（軽微な侵襲・侵襲）／侵襲を伴わない
③その他の研究（質的研究：これには人に関するグラウンデッド・セオリーや構造化面接等が含まれます）
公表予定（学会名等記載）
〔日本抗加齢医学会総会　　　　　　〕

２．課題名
還元型コエンザイムQ10含有グミの口腔機能に与える効果の検討

３．研究分担者名

氏名　斎藤一郎	所属 歯学部病理学講座	職名　教授
氏名　矢原 均	所属 株式会社カネカ	職名　常務理事　QOL 事業部長
氏名　松尾和彦	所属株式会社カネカテクノリサーチ	職名　分析部長兼開発グループリーダー
氏名　大川 勝	所属　ＣＰＣＣ株式会社	職名　代表取締役
氏名　坂野克久	所属　ＣＰＣＣ株式会社	職名　研究開発本部長

４．研究の目的と概要
＜目的＞ コエンザイムQ10（CoQ10）含有グミを摂取することによる、唾液分泌量や唾液中の酸化ストレス度・抗酸化力、嚥下機能、咬合力などの口腔機能に対する効果について解析・評価することを目的とする。
＜概要＞ CoQ10は、生命活動を維持するために必要なエネルギーの産生に必須であると同時に、ミトコンドリアの電子伝達系で発生する活性酸素に対する抗酸化剤としての役割が数多くの論文で報告されている。既に、うっ血性心不全の医薬品として販売され、今では保健機能性食品としても広く用いられている。近年では、多系統萎縮症がCoQ10遺伝子である*CoQ2*遺伝子変異によるものとして報告され、東京大学を含めて世界的に臨床治験が行なわれている。CoQ10は化学構造式の違いにより酸化型と還元型に分類され、摂取した酸化型CoQ10は体内の還元酵素により還元型CoQ10へ変換されることで作用する。CoQ10は鰯などに含有されるため食事から得ることは可能であるが、必要量を食物から摂取することは困難で、加えて加齢に伴い還元酵素も減少することから、充分なCoQ10量は望めていない事が多い。申請者らは、既にCoQ10含有カプセルを摂取することにより唾液分泌量が増加したことを報告し、菅野らも還元型CoQ10ソフトカプセルによる口腔周囲の筋力の改善などの効果を示している。

一方、咀嚼や嚥下などの口腔機能は栄養を摂取するための機能にとどまらず、認知機能や肺炎など全身に深く関与していることが周知されている。グミはドイツ語でゴムを意味し、1920年にハンス・リーゲルが子供の噛む力が減弱していることを危惧し、口腔機能を向上するために果汁などをゼラチンで固めて作ったことが始まりである。現在、我が国は超高齢社会となり8020運動をはじめ噛むことと健康長寿の関係、厚生科学研究では「口腔保健と全身的な健康状態の関係に関する研究」が実施され、口腔機能と全身機能が相互に作用していることが明となってきた。これらのことより、本研究では還元型CoQ10を含有するグミを毎日摂取することで口腔粘膜より直接成分が吸収され、唾液分泌量や口腔機能の影響について評価することを計画した。

対象者はグミを朝食後に１日１回、2個を8週間継続摂取する。摂取前と摂取8週間後の唾液分泌量、口腔機能評価として嚥下機能、咬合力、唾液酸化ストレス度・抗酸化力、唾液CoQ10量ならびに主観的評価について比較検討する。研究実施は下記の通り、統計解析方法については以下に有効性および安全性の主要な評価項目に対する統計解析方法の概要を記す。統計解析の詳細については、統計解析責任者が解析計画書に記載し、解析データ固定前に確定する。

<u>研究デザイン</u>：プラセボ対照二重盲検並行群間比較試験

<u>研究対象者</u>　：唾液分泌減少を自覚する40歳以上80歳以下の男女50名

割付け　　　：

1)試験食品の割付け

被験者割付責任者は、Microsoft Excelの乱数関数〔RAND ()〕を用いて、試験食品群X、Y群に対して試験食品A、Bを割付ける。割付ける方法として試験食品群X、Y群に乱数を発生させ、乱数の大きい順から試験食品A、Bを順に割付け、試験食品割付表（キーコード）を作成する。

2)試験食品の割当て

試験食品割付責任者は、スクリーニング検査により本試験参加が適格であると判断された被験者を対象に、唾液量を考慮して試験食品群X、Y群に無作為に割当て、試験食品と被験者識別コードを付した割当表を作成する。なお、層別因子において試験食品群間で有意差を示さないようにする。

3)試験食品割付表の封印

試験食品割付責任者は、上記1)および2)の作業終了後、改めて全ての確認を行い、問題のないことを確認し、試験食品割付表を封印する。また、試験終了まで施錠できる書庫にて開封時まで厳重に保管する。

4)試験食品割付表の開封

症例固定の終了後、試験食品割付表の開封を行う。

試験食品　　：

名称）還元型CoQ10 55mg含有食品と還元型CoQ10非含有食品

還元型CoQ10含有食品（被験食品2粒（5 g）あたり）

原材料	配合量
マルチトール	3470 mg
食物繊維	780 mg
ゼラチン	370 mg
還元型CoQ10	110 mg
グリセリン	80 mg
ビタミンC	75 mg

還元型CoQ10非含有食品（対照食品2粒（5 g）あたり）

原材料	配合量
マルチトール	3470 mg
食物繊維	780 mg
ゼラチン	370 mg
植物油脂	110 mg
グリセリン	80 mg
ビタミンC	75 mg

試験食品の用法用量：

試験食品を1日1回、2個のグミを朝食後に摂取する。

食べ忘れた場合は、翌日に2日分摂取する。ただし、1日あたりの摂取量は2日分（還元型CoQ10として220 mg）までとし、3日分以上を摂取しない。

被験者の選択および除外基準　：

自由意思により試験への参加を表明したもので、下記の選択基準を満たし除外基準に抵触しない者を対象とする。

1）選択基準

（1）唾液分泌の減少を自覚する者

（2）同意取得時の年齢が40歳以上80歳以下の成人男女

（3）事前に当該臨床試験の説明を受け、その内容が理解でき趣旨に賛同し本人による文書同意が得られる者

2）除外基準

選択基準を満たす人のうち以下を除外する。

（1）薬物や頭頸部における放射線療法による継続的な治療を行っている者

（2）試験結果に影響する可能性のあると思われる医薬品、特定保健用食品、健康食品等を常食している者

（3）心臓、肝臓、腎臓、消化器等に重篤な疾患の罹患歴および現病歴がある者

（4）試験期間中、妊娠中、授乳中、またはその希望がある者。

（5）シェーグレン症候群に罹患している者

（6）食生活が極度に不規則な者、交代制勤務者・深夜勤務者、極度なストレス状態に陥る者など生活リズムが不規則な者

（7）医薬品および食物（特にリンゴ、ゼラチン）にアレルギーがある者

（8）他の医薬品または健康食品の臨床試験に参加中、試験終了後4週間以内の者、あるいは本試験の参加

同意後に他の臨床試験に参加する予定のある者

（9）その他、試験責任歯科医師または試験分担歯科医師が本試験への参加が不適当と判断した者

検査項目と方法　：

1）　背景調査*1：性別、生年月日、年齢、既往歴、合併症（自他覚症状）、使用医薬品名、常用している医薬品・特定保健用食品・健康食品

2）　唾液量検査：14時から16時の間に安静時テスト、サクソンテスト、ガムテストを実施する。

安静時唾液はリラックスして15分間自然に分泌される唾液量を測定。

サクソンテストは滅菌ガーゼを2分間咀嚼し分泌した唾液を測定。

ガムテストはガムを10分間咀嚼し分泌した唾液を測定。

3）　嚥下機能検査(RSST：反復唾液嚥下テスト)：30秒間に自己の唾液を飲み込む回数を測定。

4）　咬合力検査：オクルーザルフォースメーターGM10を用いて臼歯部最大咬合力の測定。

5）　唾液酸化ストレス度・抗酸化力：アークレース社のポットケムi-Pack Oxystress Testは全自動即時検査用装置で唾液を用いて簡易に体内の酸化ストレス・抗酸化力を測定する。チオシアン酸化合物と鉄(III)イオンとを含む反応液は、前記両者により赤色の錯体が形成されるため、前記反応液は赤色を呈する。この反応液について、さらに還元物質を共存させると前記還元物質の還元作用（抗酸化作用）により、前記錯体を構成する鉄(III)イオンが鉄(II)イオンに還元され反応液の赤色が消失する。そこで、前記赤色を呈する反応液に前記抽出サンプルを共存させ吸光度変化量を測定することにより、唾液サンプルの抗酸化力を算出する。

6）　唾液CoQ10量：検査はカネカテクノリサーチにてLC-MS/MSを利用し微量成分を定量分析する。

7）　アンケート：抗加齢QOL問診票(別紙参照)を用いる。

*1：摂取前検査時のみ

統計解析方法　：結果は2 way repeted mesures ANOVA を用いて分析する。

解析データの固定は試験終了後に試験責任歯科医師が行う。

※なお、本研究は試験委託会社である株式会社カネカより試験実施を鶴見大学歯学部病理学講座とＣＰＣＣ株式会社に分割し、検査項目の唾液 CoQ10 量測定は株式会社カネカの関連会社である株式会社カネカテクノリサーチで実施する。加えて、ＣＰＣＣ株式会社が運営する千代田パラメディカルクリニックは、ＣＰＣＣ株式会社より依頼を受け、同クリニックで倫理審査が行われ、本研究は承認されたものである。

検査スケジュール

項目	説明会	試験食品摂取期間	
		摂取前	摂取8週後
文書同意取得	○		
施設訪問	○	○	○
被験者背景調査	○		
試験食品摂取		←→	←→
問診		○	○
唾液量検査		○	○
嚥下機能検査		○	○
唾液中抗酸化活性		○	○
アンケート		○	○

５．研究の対象と実施場所、研究期間

研究の対象　：臨床試験の主旨を理解し自発的に参加の意思を示し、唾液分泌の減少を自覚する 40 歳以上 80 歳以下の男女 50 名。参加の了承を得て、資料ならびに採取した検体を研究材料として使用する旨は文書を用いて説明し、書面で同意を得た対象者の検体のみを分析対象とする。

実施医療機関　：鶴見大学歯学部附属病院　ドライマウス外来

CoQ10 測定機関として株式会社カネカテクノリサーチ

研究期間　：倫理委員会承認後～2017 年 3 月

６．研究における医学歯学上の倫理的配慮について

（１）目的と意義を明確にし、研究により生じる危険性と医学歯学上の成果を総合判断する

＜目的と意義＞還元型 CoQ10 含有グミ咀嚼トレーニングの実施により、科学的エビデンスをもってグミを咀嚼することによる唾液腺、表情筋や咀嚼筋などの口腔機能に与える効果や心理面への影響が向上することを明らかにする。

＜研究遂行上の危険＞ 本研究における試料は唾液であり、採取は非侵襲的で危険を伴わない。

＜得られる医学上の成果＞ 既に販売されている商品に対して、含有成分である還元型 CoQ10 を口腔粘膜から直接摂取することや、食品がグミという形態であり、グミを摂取することで口腔機能の向上が認められれば、将来、本疾患発症の可能性や予防などにつながり、社会に貢献出来ると考えられる。

＜総合的判断＞ 本研究の計画を総合的に判断すると、既存商品で安全性は確保され、検査は簡便、加えて非侵襲的に得られる試料を用いて解析するため、意義のあるものと考えられる。

（２）研究の対象となる個人又は研究材料の提供者の人権擁護

研究対象者の個人情報は研究責任者(個人情報管理者)が管理を行う。対象者のうち希望があった場合、本人の解析結果の情報のみ提供する。解析結果を論文などで公表する際には、対象者の個人を識別できる情報は公表しない個人情報保護のため、検体は符号により連結可能匿名化とし、符号を結びつける対応表および個人情報は 厳重に鍵のかかる書庫に保管する。

（３）研究対象者又は提供者から同意を得る方法

研究担当者が説明文書を使用して参加者に説明し、本研究への参加について対象者から書面にて同意を得る。なお、本研究における試料・情報は、連結可能匿名化して利用する。

（４）その他

本研究はグミを無償提供する民間企業（株式会社カネカ）とは利益相反関係にあり、鶴見大学歯学部利益相反委員会の承認を得て、利害関係の公正性を保っている。受託研究費とグミや唾液 CoQ10 量測定の無償提供を受け、グミに対する製品補償は企業へ一任する。

利益相反に関して、研究結果の学会発表、論文発表などの際には所定の様式に従い開示を行う。

別紙様式３（第１３条関係）

審　査　結　果　通　知　書

平成２８年１２月１５日

病理学講座
　講師　梁 洪淵　殿

鶴見大学
学長　伊　藤　克　子　㊞

受付番号：１４２８
課　題　名：還元型コエンザイム Q10 含有グミの口腔機能に与える効果の検討
実施責任者：病理学講座　講師　梁　洪　淵

先に倫理審査申請のあった上記課題に係る研究計画、公表予定を平成２８年１１月１５日の倫理審査委員会で審査し、下記のとおり判定したので通知します。

記

判　定	承　認　　条件付承認（○）　　変更の勧告<br>不承認　　非該当
理由又は勧告	指摘事項を条件とし、条件付承認とする。<br>・申請書４の研究対象者の『健康な』を省くこと。他様式についても同様に修正すること。<br>・申請書４の除外基準を修正すること。他様式についても同様に修正すること。<br>・申請書４の検査項目と方法の２）にテスト時間を明記すること。他様式についても同様に修正すること。<br>・申請書５の研究の対象の文章中の『健康な』を省くこと。他様式についても同様に修正すること。<br>・申請書５の研究期間は倫理審査委員会承認後～とすること。他様式についても同様に修正すること。<br>・申請書６の（４）その他について、利益相反関係を詳細に明記すること。<br>・研究計画書の試験概要の試験食品について、還元型 CoQ10 を含有しない食品については『55mg』を省くこと。他様式についても同様に修正すること。<br>・研究計画書１３の原資料及び記録等の取り扱いについて、データの保存期間を終了後、５年から１０年に変更すること。<br>・同意書の代諾者欄を削除すること。

注意事項：申請受付は毎月末までとする。

Attachment 1 (Related to Article 10)

Application for Ethical Review

October 5, 2016

To the President of Tsurumi University

Investigator
Organization: Department of Pathology, School of Dental Medicine
Title: Lecturer
Name: Koufuchi Ryo Seal

*Reference ID

1. Study under application

Study design (Circle the relevant option)

(1) Interventional study / Observational study

(2) Invasive (Slightly invasive / Invasive) / Not invasive

(3) Other study (Qualitative study: It includes structured interviewing of human subjects)

Publication plan (Describe the name of the academic society)

[Scientific Meeting of the Japanese Society of Anti-Aging Medicine]

2. Study title

Investigation of the effects of chewing Gumi candy containing reduced coenzyme Q10 on oral function

3. Subinvestigators

Name	Kazuo Saito	Organization	Department of Pathology, School of Dental Medicine	Title	Professor
Name	Hitoshi Yahara	Organization	Kaneka Corporation	Title	Board member, Head of QOL business
Name	Kazuhiko Matsuo	Organization	Kaneka Techno-Research, Co. Ltd.	Title	Head of Analysis, Development Group Leader
Name	Masaru Okawa	Organization	CPCC Co., Ltd.	Title	Representative President
Name	Katsuhisa Sakano	Organization	CPCC Co., Ltd.	Title	Head of R&D

4. Study purpose and outline

<Purpose> To analyze and evaluate the effects of chewing Gumi candy containing Coenzyme Q10 (CoQ10) on the amount of saliva secreted, level of oxygen radicals in saliva, antioxidant capacity of saliva, swallowing function, and bite force.

<Outline> There have been many reports that CoQ10 is essential to produce the energy required for maintenance of life and that it acts as an antioxidant for oxygen radicals in the mitochondrial electron transmission system. It is already employed to treat congestive heart failure and is widely used in health foods. Recently, multiple system atrophy was reported to be caused by mutation of the *CoQ2* gene, which is one of the CoQ10 genes. Clinical studies are being conducted around the world, including at the University of Tokyo. CoQ10 is classified into oxidized and reduced types depending on its chemical structure. Oxidized CoQ10 is ingested and converted to reduced CoQ10 by reducing enzymes, after which it exerts its effects. CoQ10 is contained in rice and is available via the diet. However, it is difficult to obtain the necessary amount from the diet alone. Moreover, reducing enzyme activity decreases as people age. Thus, deficiency of CoQ10 often occurs. The applicant has already reported that intake of capsules containing CoQ10 increased the production of saliva. Also, Sugano et al. demonstrated that intake of soft capsule containing reduced CoQ10 improved the strength of muscles around the oral cavity.

It is well known that oral functions such as mastication and swallowing are not only required for intake of nutrients, but are also influence systemic functions such as cognitive ability or the development of pneumonia. “Gumi” means rubber in German. In 1920, Dr. Hans Riegel was concerned that the chewing ability of children was decreasing, and prepared Gumi candy by solidifying fruit juice with gelatin in order to improve oral function. Currently, super aging of society is occurring in Japan. In addition to *Promotion 8020* (a campaign encouraging Japanese people to keep 20 or more of their own teeth until the age of 80), there are other activities focusing on the relationship between healthy longevity and chewing, such as the Ministry of Health, Labour and Welfare (MHLW) Grant for Scientific Research on “Investigation of the relationship between oral health and general health.” As a result, it is now known that oral function is closely related to systemic functions. Consequently, we have planned a study to investigate whether daily chewing of Gumi candy containing reduced CoQ10 results in direct absorption of CoQ10 through the oral mucosa, increases the production of saliva, and affects oral function.

The subjects will chew 2 pieces of Gumi candy once daily after breakfast for 8 weeks. The following comparisons will be made between before the study and after 8 weeks: amount of saliva secreted, oral functions such as swallowing and bite

force, level of oxygen radicals in saliva, antioxidant capacity of saliva, salivary level of CoQ1, and subjective assessments. The study procedures are shown below. An outline of statistical analysis of the primary efficacy and safety endpoints is provided below. Details of statistical analysis will be described in the Statistical Analysis Plan written by the statistician and will be finalized prior to database locking.

Study design: Placebo-controlled, double-blind, parallel group comparative study.

Study subjects: 50 men and women aged between ≥40 and ≤80 years with a subjective decrease in secretion of saliva.

Randomization:

1) Randomization of study products
   The study randomization manager will use the random number function of Microsoft Excel [RAND ()] to randomize the study products A and B to groups X and Y. The randomization method is as follows: Random numbers will be generated for study groups X and Y, and the study products A and B will be assigned in descending order of these numbers. Then the study product randomization table (key code) will be prepared.
2) Allocation of study products
   The study randomization manager will randomly assign subjects to groups X and Y if they are judged to be eligible based on the results of the screening test and their secretion of saliva. An assignment table describing the study product and the subject ID nos. will be prepared. There should be no significant differences between the study groups for each stratification variable.
3) Sealing of the study product assignment table
   Following completion of 1) and 2) above, the study randomization manager will check everything and confirm that there are no issues. Then the study product assignment table will be sealed and it will be securely stored in a locked cabinet until study completion.
4) Opening of the study product assignment table
   After database locking, the study product assignment table will be opened.

Study product:

Name) Gumi candy without or with reduced CoQ10 (55 mg)

Candy containing reduced CoQ10 (per 2 pieces of the test candy (5 g))

Raw materials	Composition
Maltitol	3470 mg
Dietary fiber	780 mg
Gelatin	370 mg
Reduced CoQ10	110 mg
Glycerin	80 mg
Vitamin C	75 mg

Candy not containing reduced CoQ10 (per 2 pieces of the control candy (5 g))

Raw materials	Composition
Maltitol	3470 mg
Dietary fiber	780 mg
Gelatin	370 mg
Plant oil	110 mg
Glycerin	80 mg
Vitamin C	75 mg

Dosage and administration of the study product:

Two (2) pieces of candy will be chewed after breakfast once daily.

If a dose is missed, double the amount is taken on the next day. However, the maximum daily dose is limited to the amount for two days (220 mg of reduced CoQ10), and the amount for 3 days or more is not to be taken.

Inclusion and exclusion criteria:

Subjects who voluntarily give consent to participation in the study and satisfy the following inclusion criteria without meeting any exclusion criteria.

1) Inclusion criteria
   (1) Persons who have noted a subjective decrease in the secretion of saliva.
   (2) Men and women aged between ≥40 and ≤80 years at the time of giving informed consent.
   (3) Persons who have received an explanation of this clinical study, and who are able to understand the study and give written consent themselves.
2) Exclusion criteria
   Among candidate subjects who meet the inclusion criteria, the following persons are excluded.
   (1) Patients receiving pharmacotherapy or radiation therapy to the head and neck.
   (2) Persons who regularly use drugs, specified health foods, or health foods that could affect the results of the study.
   (3) Patients with a history of (or currently suffering from) severe diseases affecting the heart, liver, kidneys, or digestive tract.
   (4) Pregnant women, nursing women, or women who wish to become pregnant during the study period.
   (5) Patients with Sjogren syndrome.

(6) Persons with extremely irregular dietary habits, as well as those on shift work or night shift, and those with an irregular lifestyle such as those under severe stress.
(7) Persons with allergies to drugs and foods (especially apple and gelatin).
(8) Persons currently participating in another clinical study of a drug or health food, persons who completed such a study within 4 weeks before enrolment, or persons who intend to participate in another study after consenting to this study.
(9) Other persons whom the investigator or subinvestigator judges to be inappropriate for this study.

Test items and methods

1) Demographic profile*1: Sex, date of birth, age, medical history, complications (symptoms/signs), current medications, regular drugs, specified health foods/health foods.
2) Measurement of the saliva flow rate: A rest test, Saxon test, and gum test will be conducted from 14:00 to 16:00.
   The rest test measures spontaneous secretion of saliva over 15 minutes when a subject is relaxed.
   The Saxon test measures secretion of saliva when sterile gauze is masticated for 2 minutes
   The gum test measures secretion of saliva when gum is masticated for 10 minutes.
3) Swallowing function test: repeated saliva swallowing test (RSST). The number of times saliva is swallowed during a 30-second period is measured.
4) Bite force test: A GM10 occlusal force meter is used to measure the maximum bite force of the molars.
5) Salivary level of oxygen radicals/antioxidant capacity: The Pocket Chem i-Pack Oxystress Test (Arc race) is an automated rapid test used to measure oxidative stress/ antioxidant capacity. The reaction solution contains thiocyanic acid compounds and iron (III) ions that form a red complex with oxygen radicals, so the reaction solution becomes red in the presence of radicals. If a reducing substance is added, the reducing effect (antioxidant effect) of the substance will reduce iron (III) ions to iron (II) ions and the reaction solution will lose its red color. Accordingly, an extract of saliva is added to the aforementioned reaction solution and the change of absorbance is measured. Then the antioxidant capacity of saliva samples is calculated.
6) Amount of CoQ10 in saliva: Trace components of samples are quantitated by LC-MS/MS at Techno-Research at Kaneka.
7) Questionnaire: The Anti-aging QOL questionnaire (refer to the Attachment) will be used.

*1: Only for the test before ingestion

Statistical analysis: The results will be analyzed by 2-way ANOVA with repeated measures.
The investigator will lock the analysis data after study completion.

* The sponsor (Kaneka Corporation) will outsource this study to the Department of Pathology, School of Dental Medicine, Tsurumi University and CPCC Co. Ltd. Assay of CoQ10 in saliva samples will be conducted at Kaneka Techno-Research, a subsidiary of Kaneka Corporation. In addition, Chiyoda Paramedical Clinic (managed by CPCC Co. Ltd.) was requested to participate by CPCC, after which ethical review was conducted at the clinic and approval was granted.

Examination schedule

Item	Explanation meeting	Study product ingestion period	
		Before ingestion	Week 8
Written informed consent	○		
Site visit	○	○	○
Subject demographic profile	○		
Study product ingestion		◄──────────	──────────►
Medical check		○	○
Measurement of saliva		○	○
Swallowing function test		○	○
Salivary antioxidant activity		○	○
Questionnaire		○	○

5. Study subjects, study site, and study period

Study subjects: Fifty (50) men or women aged between ≥40 and ≤80 years who understand the purpose of the clinical

study, voluntarily indicate willingness to participate in the study, and have the subjective symptom of decreased secretion of saliva. An explanation will be provided by using the Subject Explanation Sheet before obtaining consent, and only samples obtained from subjects who consent will be analyzed.

Study site: Dry Mouth Clinic, Tsurumi University Dental Hospital
Kaneka Techno-Research, Co. Ltd. for assay of CoQ10

Study period: After approval by the Ethics Committee until March 2017

6. Ethical considerations from the perspective of medical and dental science

(1) Clarification of the study purpose and significance, and comprehensive evaluation of the risks associated with the study and its medical and dental benefits

<Purpose and significance> By regular mastication of Gumi candy containing reduced CoQ10, scientific evidence will be obtained regarding its effects on the function of oral structures such as the salivary glands, facial muscles, and masticatory muscles, as well as the psychological effects of mastication.

<Risks associated with the study> This study uses saliva samples and sampling is noninvasive. Accordingly, there are no risks.

<Medical and dental benefits expected> It will be determined whether reduced CoQ10 in Gumi candy (a commercial product) can be directly absorbed through the oral mucosa. In addition, if chewing Gumi candies improves oral function, it may lead to prevention of oral disease and thus contribute to society.

<Overall assessment> Taking into consideration the overall program of this study, safety is ensured since a commercial product is being used. Also, testing is convenient and samples are obtained in a noninvasive manner. Therefore, this study is considered to be useful.

(2) Protection of the rights of subjects

Personal information of the subjects will be retained by the investigator (Personal Information manager). If a subject wishes, the results of analysis will only be provided to that subject. When the results are published, any information that identifies an individual will not be disclosed in compliance with Personal Information Protection. A linked anonymized ID number will be assigned to each sample, and the correspondence table and personal information will be stored in a secure locked cabinet.

(3) Method of obtaining consent from subjects

Study staff will give an explanation to a candidate subject using the Subject Information Sheet and obtain written informed consent. Anonymized linking of samples and information will be done in this study.

(4) Other matters

There is a conflict of interest in relation to a private company (Kaneka Corporation) providing Gumi candy free of charge. However, the Conflict of Interest Committee of the Tsurumi University School of Dental Medicine determined that this conflict of interest can be managed. Research funds, Gumi candy for use in the study, and assay of CoQ10 in saliva will be provided free of charge by Kaneka Corporation and Gumi product liability will be the responsibility of the company. Regarding conflict of interest, the specified form shall be followed when presenting the study results at a conference or at the time of publication.

Attachment 3 (Related to Article 13)

Notification of Review Result

December 15, 2016

To Koufuchi Ryo, Lecturer

Department of Pathology

Tsurumi University
Katsuko Ito, President Seal

Reference ID: 1428
Study title: Investigation of the effects of chewing Gumi candy containing reduced coenzyme Q10 on oral function
Investigator: Koufuchi Ryo, Lecturer, Department of Pathology

Regarding the above study for which Ethics Committee review was requested, the protocol was reviewed by the Ethics Committee on November 15, 2016. Please find the judgment below.

Note

Judgment	Approval / (Conditional approval) / Recommendation for change / Not approved / Not applicable
Reason or recommendation	This application is conditionally approved with the following observations.<br>• In Application 4, "Healthy" study subjects. "Healthy" should be deleted. The same applies elsewhere.<br>• In Application 4, the Exclusion criteria should be modified. The same applies elsewhere.<br>• In Application 4, Test items and methods 2), the test time should be described. The same applies elsewhere.<br>• In Application 5, Study subjects, "Healthy" should be deleted. The same applies elsewhere.<br>• In Application 5, Study period, the description should be changed to "After approval by the Ethics Committee until ...." The same applies elsewhere.<br>• In Application 6, (4) Other matters, the conflict of interest should be described in detail.<br>• For the study product in Study Outline of the Study Plan, "55 mg" should be deleted from the product not containing reduced CoQ10. The same applies elsewhere.<br>• In Study Plan 13, Handling of Source Documents and Records, the data storage period should be changed from 5 years to 10 years.<br>• The term Legal Representative should be deleted from the section about Informed Consent.

Note: Applications must be submitted by the end of each month.
